# Molecular Dynamics Study of Binding of *µ*-Conotoxin GIIIA to the Voltage-Gated Sodium Channel Na_v_1.4

**DOI:** 10.1371/journal.pone.0105300

**Published:** 2014-08-18

**Authors:** Somayeh Mahdavi, Serdar Kuyucak

**Affiliations:** School of Physics, University of Sydney, Sydney, New South Wales, Australia; Dalhousie University, Canada

## Abstract

Homology models of mammalian voltage-gated sodium (Na_V_) channels based on the crystal structures of the bacterial counterparts are needed to interpret the functional data on sodium channels and understand how they operate. Such models would also be invaluable in structure-based design of therapeutics for diseases involving sodium channels such as chronic pain and heart diseases. Here we construct a homology model for the pore domain of the Na_V_1.4 channel and use the functional data for the binding of *µ*-conotoxin GIIIA to Na_V_1.4 to validate the model. The initial poses for the Na_V_1.4–GIIIA complex are obtained using the HADDOCK protein docking program, which are then refined in molecular dynamics simulations. The binding mode for the final complex is shown to be in broad agreement with the available mutagenesis data. The standard binding free energy, determined from the potential of mean force calculations, is also in good agreement with the experimental value. Because the pore domains of Na_V_1 channels are highly homologous, the model constructed for Na_V_1.4 will provide an excellent template for other Na_V_1 channels.

## Introduction

Voltage-gated sodium (Na_V_) channels are responsible for initiation and propagation of action potential, which is essential for the activity of excitable cells such as neurons, heart and muscle cells [1]. Na_V_ channels are involved in many physiological activities throughout the body, which also makes them potential drug targets for related disorders such as cardiac and neuropathic diseases [2]–[5]. Because of their prominence, much effort has gone into understanding the structure and function of Na_V_ channels. For a very long time, no molecular structures were available for Na_V_ channels, and the low resolution structures obtained from cryo-electron microscopy [6] were not very informative. In the absence of crystal structures, indirect methods such as studying the effect of mutations on ligand binding [7]–[12] have been the main source of information on Na_V_ channels.

This situation has changed dramatically with the recent determination of the crystal structures of several bacterial Na_V_ channels [13]–[17]. As in potassium channels, where solution of the bacterial K^+^ channel KcsA [18] started an intense period of examination of the structure-function relations, we expect a similar thing to happen in Na_V_ channels. Already, there have been many computational studies of the bacterial Na_V_ channels, investigating their ion permeation [19]–[27] and gating [28], [29] properties, as well as ligand binding [30], [31]. There are, as yet, no crystal structures for the mammalian Na_V_ channels. Unlike in potassium channels, the tetrameric symmetry is lost when going from the bacterial to the mammalian Na_V_ channels. Therefore, solution of the structures of the mammalian Na_V_ channels is likely to be more difficult and may not follow soon. This leaves construction of homology models from the bacterial ones as the most viable route for making progress. Again this is not as straightforward as in potassium channels because, besides the loss of the tetrameric symmetry, the critical selectivity filter is not conserved either between the bacterial and the mammalian Na_V_ channels. Nevertheless, the bacterial Na_V_ channels provide a reasonable scaffold for construction of homology models for the mammalian ones, and such models can be constrained and validated using the large amount of functional data that have been accumulated over several decades [1]. Initial attempts in this regard include a docking study of tetrodotoxin and anesthetics binding to the pore domain of the Na_V_1.4 channel [32], and an MD study of Na^+^/K^+^ selectivity in Na_V_1 channels [33].

Na_V_ channels are targets for many toxins, which bind with high affinity to various sites on the channel protein, disabling its normal function. For example, tetrodotoxin, saxitoxin, and *µ*-conotoxins bind to the channel vestibule and block the pore [1], [34]. This has been exploited in many experimental studies, where the known toxin structures were used to probe the pore domain of Na_V_ channels [35], [36]. Because *µ*-conotoxins are the only peptide toxins that block Na_V_ channels, there has been a lot of interest in their properties. The first *µ*-conotoxin to be characterised was *µ*-conotoxin GIIIA (*µ*-GIIIA), which selectively binds to the Na_V_1.4 channel [37], [38]. Its structure was determined from NMR by several groups [39]–[41]. A great deal of mutagenesis and functional studies have been performed on the Na_V_1.4–*µ*-GIIIA complex to determine the binding mode and identify the key residues involved in binding [42]–[55]. For this reason, Na_V_1.4–*µ*-GIIIA complex provides a unique system for testing the homology models of mammalian Na_V_ channels that are constructed from the bacterial ones.

Here, we construct a homology model for the pore domain of the Na_V_1.4 channel based on the crystal structure of Na_V_Ab. The stability of the Na_V_1.4 model is checked via molecular dynamics (MD) simulations. We then create a model for the Na_V_1.4–*µ*-GIIIA complex using the docking program HADDOCK [56], [57], followed by refinement in MD simulations. The proposed binding mode for the Na_V_1.4–*µ*-GIIIA complex is shown to give a satisfactory account of the available mutagenesis data. As a dynamical test of the complex model, we have determined the binding free energy of *µ*-GIIIA from its potential of mean force (PMF), which is again found to be in good agreement with the experimental value. The same computational methodology has previously been used to study toxin binding to potassium channels, where its ability to yield accurate protein-ligand complexes and binding free energies has been demonstrated [58]–[64]. The present study extends it to sodium channels, where much more work remains to be done.

## Methods

### Modeling of Na_V_1.4 Channel

**Figure 1:**
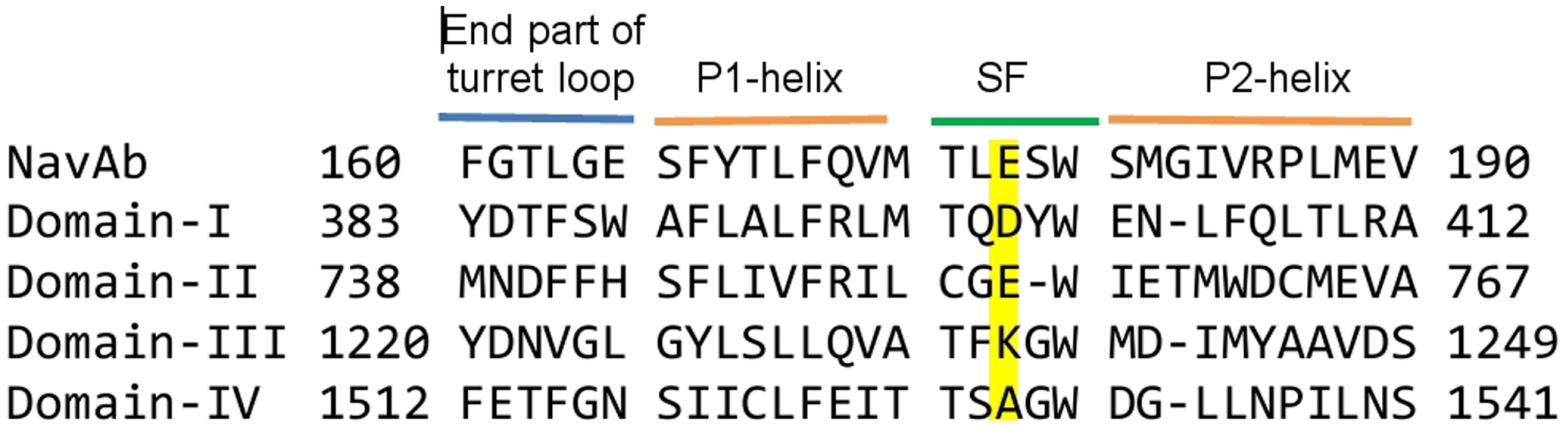
Alignment used in homology modeling of rNa_v_1.4. The DEKA residues in the four domains forming the selectivity filter (SF) are highlighted.

We construct a homology model for the pore domain of Na_V_1.4 using the crystal structure of Na_V_Ab (PDB ID: 3RVY) [13]. The sequence of Na_V_1.4 is taken from the Uniprot database (P15390). As shown in Figure 1, the sequences for the four domains of Na_V_1.4 are all quite different from that of Na_V_Ab. This makes homology modeling of Na_V_1.4 not so straightforward as has been noted earlier [32]. Here, we have aligned the critical (DEKA) residues in the selectivity filter with the corresponding (EEEE) residues in 3RVY. A gap is placed between the E and W residues in domain II of the selectivity to align the conserved W residues. Then, we do multiple alignments between S5-pore-S6 sequence for all four domains of Na_V_1.4 and Na_V_Ab using the program ClustalW [66]. The final alignments obtained for the P1-SF-P2 sequences are shown in Figure 1, which are the same as those in [15] but differ from the ones in [32] in the placement of the gap in domains I, III, and IV, which is after the W residues in the selectivity filter.

While acceptable alignments are obtained for the pore domain, it is much harder to do the same for the S5-P1 and P2-S6 linker sequences in the turret because there are no good templates, and much less data are available on their function. Therefore, we have neglected these linker sequences in our current model of Na_V_1.4. While the S5-P1 linker faces the pore, it does not appear to be involved in binding of *µ*-GIIIA, hence our results are unlikely to be affected by its absence. A 3D model of the channel is created using Modeller [67] by threading the aligned Na_V_1.4 sequence for each domain on a corresponding domain of 3RVY. In order to refine the model and check its stability, we have performed MD simulations of the Na_V_1.4 model in a membrane environment. For this purpose, we have used the protocols established in previous MD simulations of ion channels [68], [69]. The Na_V_1.4 model is embedded in a lipid bilayer consisting of 153 POPE molecules in the x-y plane and solvated with a 0.1 M NaCl solution. Extra counter ions are included in the system to neutralize it where necessary. The system is then equilibrated in MD simulations in several stages. First the protein is fixed and the system is equilibrated with pressure coupling until the correct water and lipid densities are obtained. At that point, the x and y dimensions of the simulation box are fixed and pressure coupling is applied only in the z direction (the box size is about 95×96×84 Å^3^). In the second stage, the restraints on the protein atoms are relaxed gradually by first reducing those on the side chain atoms from 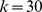 kcal/mol/Å^2^ to 0 in 3 ns. Finally, the backbone atoms are relaxed in a similar manner. The resulting system is run for 0.1* µ*s to check the stability of the model. The rmsd of the backbone atoms of Na_V_1.4 formed a plateau after the first few ns which remained stable throughout the 0.1* µ*s of MD simulations with an average value of 0.6 Å, confirming its stability.

### Modeling of Na_V_1.4–*µ*-GIIIA Complex

**Figure 2:**
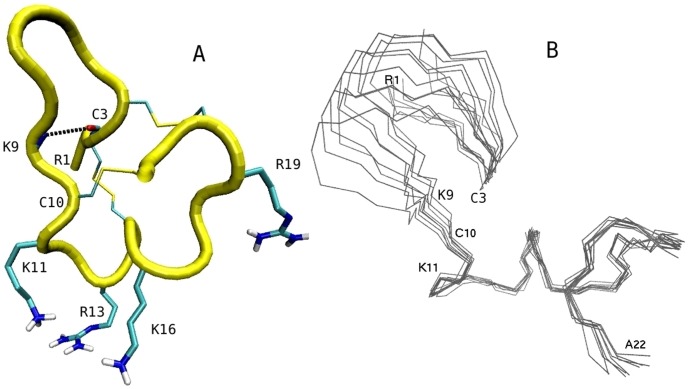
NMR structures of *µ*-GIIIA. (A) Structure of *µ*-GIIIA with the pore blocking residues K11, R13 and K16 pointing downward. Three disulfide bridges and the C3–K10 hydrogen bond stabilizing the structure are indicated explicitly. (B) Superposition of the ten NMR structures demonstrating the flexibility of the N-terminal residues 1–9 around the C3 and C10 hinges.

The conotoxin *µ*-GIIIA is a 22-residue peptide with the sequence RDCCTOOKKC KDRQCKOQRC CA with an amidated Ala at the C-terminal. It has three Arg, four Lys and two Asp residues with a total charge of +6*e*. The NMR structure of *µ*-GIIIA is shown in Figure 2A (PDB ID: 1TCJ) [41]. The side chains of the four basic residues involved in binding of *µ*-GIIIA to Na_V_1.4 are indicated explicitly in the figure. The structure of *µ*-GIIIA is stabilized by three disulfide bridges between C3–C15, C4–C20, and C10–C21. However, as seen from the superposition of the NMR snapshots in Figure 2B, the peptide has quite a bit of flexibility around the hinges at the C3 and C10 residues. For this reason, the residues 11–22 forming the binding interface will be used in the rmsd calculations when we check for distortion of the toxin during umbrella sampling MD simulations.

The initial poses for the Na_V_1.4–*µ*-GIIIA complex are generated by docking the *µ*-GIIIA structure to the Na_V_1.4 model using the program HADDOCK [56], [57]. In previous studies of toxin binding to potassium channels, HADDOCK has given very good results, reducing the time needed for refinement with MD [59]–[63], [70]. In order to get an adequate sampling of the side chain orientations, we use all ten NMR conformers of *µ*-GIIIA in ensemble docking. Because there are no well-known binding motifs for Na_V_1 channel blockers—like the pore inserting Lys in potassium channel blockers—we have considered several possibilities for restraints in HADDOCK. To facilitate comparisons with the mutation data and simplify interpretation of the results, we use a single restraint in each docking study. The EEDD and DEKA ring of residues are the potential sites on the channel for using restraints. However, the mutation data indicates that the EEDD residues play a much more important role in binding of *µ*-GIIIA than the DEKA residues [47]. Therefore, only the EEDD ring is used as a restraint site in the following docking studies. The potential restraint sites on the toxin include the residues R1, K11, R13, K16, and R19, which are identified in mutagenesis experiments (see Table 1). Separate docking studies are performed for each of these residues and the EEDD ring as a restraint. One thousand conformations are generated from each docking, and the top hundred selected on the basis of scoring are analyzed via clustering to find the dominant binding mode. In choosing among the five sets of complexes generated, we have also used the fact that *µ*-GIIIA blocks the channel. This criterion is satisfied only for the complexes in the set with the R13–EEDD restraint, which also have the highest scores. The complex structure in the top-ten of this set that has the most toxin–channel contacts is selected for refinement in MD simulations (see Table 2).

**Table 1:** Effect of the mutations in *µ*-GIIIA on the IC_50_ values for binding to Na_v_1.4.

Res.	Mut.	IC5_50_(wt)	IC_50_(mut)	Ref.
		(nM)	IC_50_(wt)	
R1	A	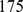	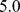	[42]
R1	A	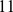	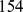	[48]
R1	A	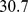	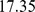	[53]
R1	K	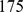	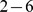	[42]
R1	Q	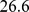	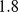	[43]
D2	A	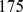	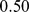	[42]
D2	N	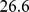	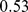	[43]
O6	P	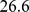	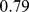	[43]
O7	P	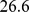	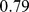	[43]
K8	A	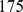	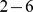	[42]
K8	A	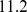	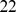	[55]
K8	Q	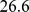	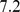	[43]
K9	A	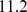	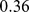	[55]
K9	Q	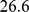	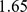	[43]
K11	A	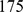	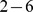	[42]
K11	A	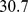	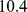	[53]
K11	A	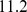	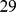	[55]
K11	Q	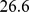	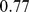	[43]
D12	A	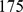	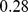	[42]
D12	A	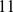	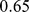	[48]
D12	N	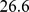	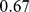	[43]
R13	A	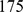	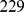	[42]
R13	A	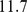	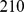	[46]
R13	A	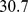	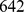	[53]
R13	K	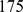	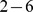	[42]
R13	K	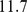	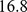	[46]
R13	Q	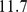	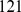	[46]
R13	D	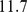	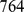	[46]
Q14	D	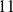	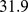	[48]
K16	A	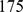	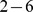	[42]
K16	A	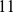	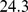	[48]
K16	A	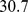	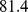	[53]
K16	Q	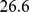	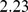	[43]
O17	P	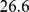	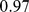	[43]
O17	P	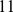	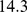	[48]
Q18	K	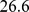	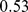	[43]
R19	A	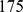	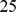	[42]
R19	A	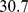	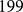	[53]
R19	A	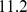	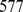	[55]
R19	K	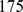	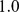	[42]
R19	Q	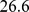	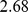	[43]

The residue and its mutation are listed in the first two columns. The experimental IC_50_ values for the wild type are given in the third column and the ratio IC_50_(mut)/IC_50_(wt) in the fourth column. The data are collected using different experimental systems (oocytes, mammalian cells or lipid bilayers) and varying ionic strengths, which partly explains the variation in the measured IC_50_ values.

**Table 2:** List of interacting residues in the Na_v_1.4–*µ*-GIIIA complex.

*µ*-GIIIA	Na_v_1.4	Dock.	MD Aver.
K8-N*_z_*	D1248-O_1_	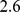	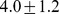
K11-N*_z_*	D1241-O_1_	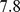	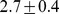
K11-N*_z_*	D1532-O_1_	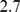	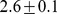
R13-N_2_	E403-O_1_	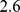	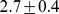
R13-N_1_	E758-O_2_	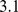	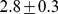
R13-N_2_	D1532-O_2_	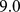	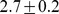
K16-N*_z_*	E758-O_1_	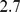	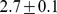
K16-N*_z_*	D1241-O_2_	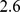	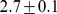
R19-N_2_	D762-O_2_	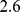	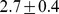

The average N–O distances obtained from HADDOCK and MD simulations are given in the third and fourth columns (in units of Å).

The complex structure obtained from docking is aligned with the channel model in the membrane, and the coordinates of the toxin are transferred to the channel model. The protocol used in equilibrating the channel protein is also used for the complex, with the channel protein and toxin being relaxed simultaneously. The equilibrated system is run for another 50 ns to check its stability and generate trajectory data for analysis. During this MD simulation, a small restraint with 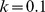 kcal/mol/Å^2^ is applied to channel backbone atoms to preserve the integrity of the channel but no restraints are imposed on the toxin. The system is found to be well-equilibrated from the start, and therefore, the trajectory data are used for the analysis of the complex structure.

### MD Simulations and PMF Calculations

All MD simulations are performed using version 2.7 of NAMD [71] with the CHARMM36 force field [72]. An NpT ensemble is used with periodic boundary conditions. Pressure is kept at 1 atm and temperature at 300 K using Langevin coupling with damping coefficients of 5 ps^−1^. Lennard-Jones interactions are switched off smoothly within distance of 10–13.5 Å. Electrostatic interactions are computed without truncation using the particle-mesh Ewald algorithm. A time step of 2 fs is employed in all MD simulations. The trajectory data is saved at 1 ps intervals but the reaction coordinate is written at every time step in umbrella sampling simulations.

The PMF for dissociation of *µ*-GIIIA from Na_V_1.4 is constructed using umbrella sampling MD simulations. As the method has been described in detail previously [58], [63], we give only a brief description here. The reaction coordinate is chosen as the distance between the center of mass (COM) of the channel protein and the COM of the toxin along the channel axis. Initially 28 umbrella windows are created along the channel axis at 0.5 Å intervals using steered MD with a force constant 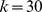 kcal/mol/Å^2^ and pulling speed 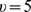 Å/ns. Six more windows are added subsequently to ensure that the toxin has reached the bulk region signalled by a flat PMF. The same force constant of 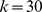 kcal/mol/Å^2^ is used in umbrella sampling simulations, which is found to be optimal for toxins of this size [58]. The overlaps of distributions between the neighboring windows should be 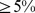 to avoid numerical instabilities in construction of the PMF. We have included two more windows between the windows 4–5 and 22–23, where this condition is not satisfied. The reaction coordinates collected from the simulations are unbiased and combined using the weighted histogram analysis method [73]. Umbrella sampling simulations are continued until the convergence of the PMF is assured from block data analysis of the PMF data.

The binding constant is determined by integrating the PMF, 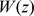, along the 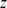 axis [58], [63]

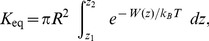
(1)where the 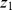 and 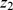 are the initial and final points in the PMF, and 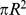 is the average cross sectional area of the binding pocket as explored by the COM of the toxin, which is determined from its transverse fluctuations. The value of 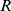 is obtained from restraint-free MD simulations of the Na_V_1.4–*µ*-GIIIA complex as 0.79 Å. Finally, the standard binding free energy of *µ*-GIIIA is obtained from the binding constant using
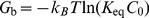
(2)where 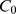 is the standard concentration of 1 M.

## Results and Discussion

### Binding Mode of the Na_V_1.4–*µ*-GIIIA Complex

**Figure 3:**
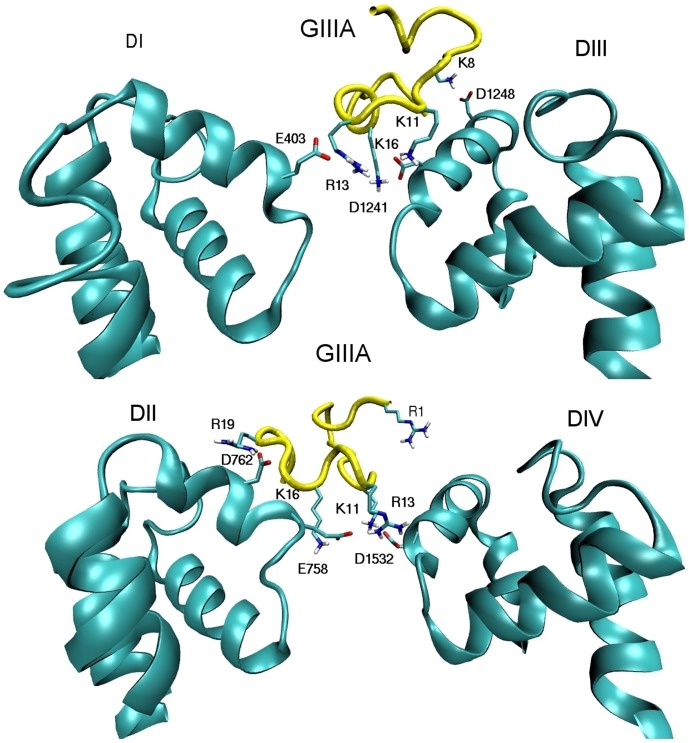
Binding mode of the Na_v_1.4–*µ*-GIIIA complex. For clarity, domains I and III (top) and II and IV (bottom) are shown separately. All the important interactions between the channel and toxin residues are indicated explicitly.

Snapshots of the Na_V_1.4–*µ*-GIIIA complex obtained from docking and MD simulations are shown in Figure 3 (A PDB file giving the coordinates of the complex structure is provided in File S2). The five basic residues on the toxin that make contacts with the acidic residues on the channel are indicated explicitly. To provide a more quantitative picture of the binding mode, we have calculated the average N–O distances between the interacting residues from the 50 ns MD trajectory data (Table 2). The distances obtained from HADDOCK are also shown for comparison. Of the nine contact pairs listed, HADDOCK has got six of them within 0.3 Å of the MD result and one of them within 1.5 Å. Only in two cases, the discrepancy is larger than 5 Å. Considering that the binding mode is rather complex involving multiple partners for some of the residues, this is an excellent result from HADDOCK. It also explains why the complex structure obtained from HADDOCK has equilibrated so quickly in MD simulations.

In order to validate the Na_V_1.4–*µ*-GIIIA complex, we compare the binding mode characterized in Figure 3 and Table 2 with the mutagenesis data summarized in Table 1. The toxin residue R13 makes the most contacts with the channel residues and hence is expected to play a major role in binding, which is in good agreement with experiments (Table 1). The R13A mutation causes more than 200-fold increase in the IC_50_ ratio. From Eq. 2, this corresponds to more than 3 kcal/mol change in the binding free energy. In previous studies of binding of ShK toxin to K_V_1 channels, [59]–[61], we have shown that neutralizing a charged toxin residue in contact with channel residues costs about 2 kcal/mol in binding free energy, provided the binding mode is preserved after the mutation. However, if the binding mode of the mutated toxin is substantially different from that of the wild type, there could be larger changes in the binding free energy. We have, therefore, performed docking calculations for *µ*-GIIIA[R13A] and found that the binding mode has completely changed with R1 inserting in the Na_V_1.4 pore. This highlights the importance of checking the binding mode when interpreting unusual results in alanine scanning experiments. Other mutations of R13 also reduce the affinity, including the conservative mutation R13K, presumably because a Lys residue cannot sustain the multiple contacts R13 makes. R13 is followed in importance by the residues R19, K16, and K11, each of which makes tight contacts with the acidic residues on the channel. For these residues, the observed loss of affinity, when they are mutated to Ala, are mostly in the range expected from switching off the charge (Table 1). The quality of the charge contact between K8 and D1248 is not as good as the others because K8 is on the flexible N-terminal part of the toxin, which fluctuates around the C10 hinge. As a result, the K8–D1248 average distance and its sigma are substantially larger compared to the other charge contacts. Again this is consistent with the experimental observation that K8A mutation causes less affinity loss relative to the other contacts listed in Table 2 (Table 1). Further evidence for the relative strength of the individual interactions will be presented when we discuss the persistence of contacts during dissociation of *µ*-GIIIA from Na_V_1.4.

The only residue whose mutation to Ala reduces the affinity of *µ*-GIIIA but does not appear in the proposed binding mode is R1. As seen from Figure 3, R1 is on the opposite side of the binding interface and does not make any contacts with the channel residues (the N–O distance for the closest acidic residue on the channel is more than 9 Å). We have seen in studies of potassium channel toxins that mutation of an Arg residue could still result in a different binding mode even though it is not directly involved in binding [59], [61]. To see if a similar thing happens in *µ*-GIIIA, we have docked *µ*-GIIIA[R1A] to Na_V_1.4. The resulting binding mode is found to be substantially different from that of *µ*-GIIIA, which indicates that the reduction in affinity is likely to be caused by a change in the binding mode rather than loss of a charge interaction with the channel residues.

**Figure 4:**
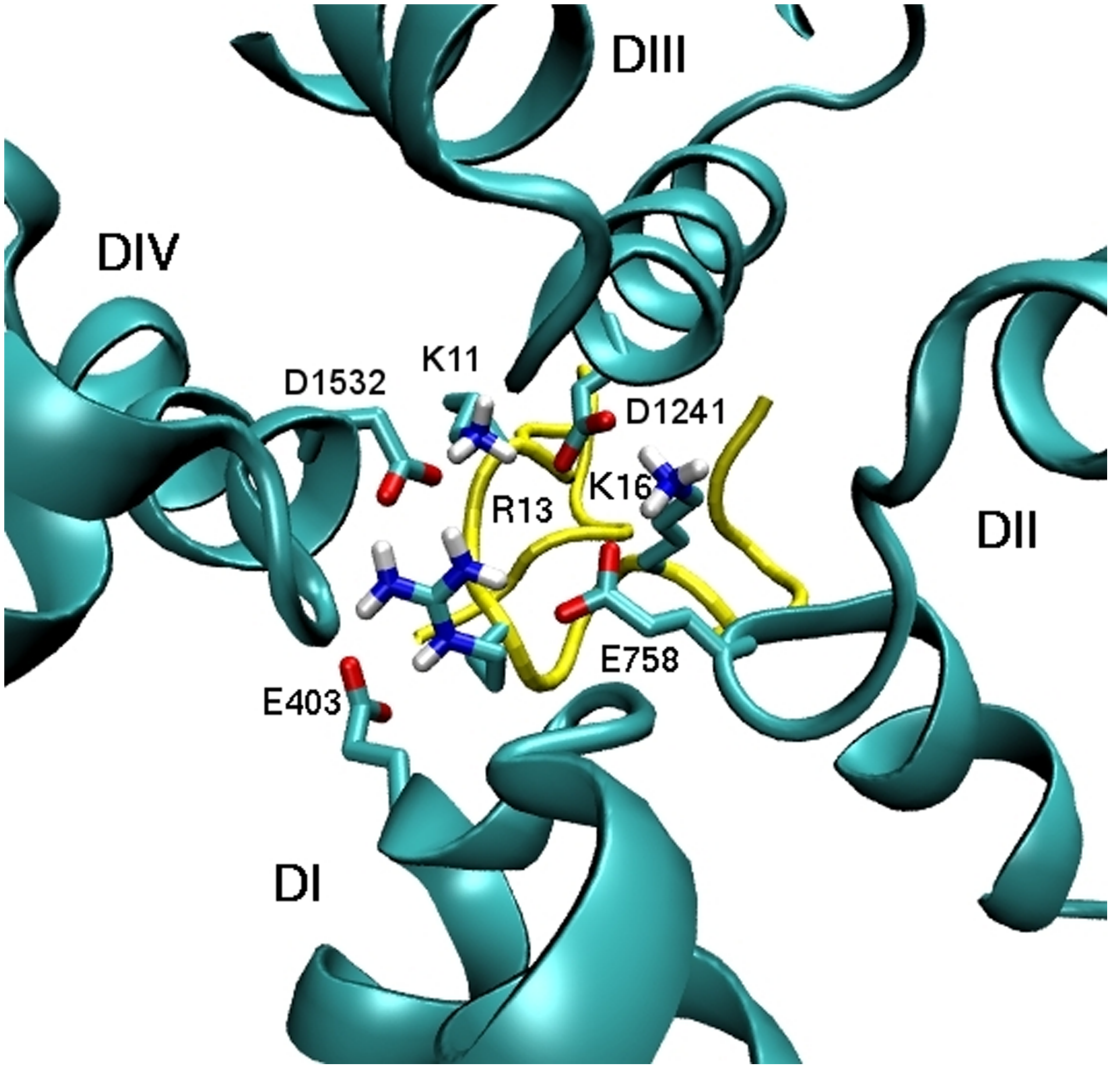
Bottom view of the complex structure demonstrating blocking of the pore. The toxin residues R13, K11, and K16 make contacts with the channel residues EEDD in all four domains.

The binding mode emerging from this study provides a complete block of the fairly large vestibule of Na_V_1.4. This is illustrated in Figure 4, which shows a bottom-view of the complex. The three basic residues, R13, K16, and K11 are seen to weave around the EEDD ring, making multiple contacts with residues in all four domains. We note that there is some redundancy here because two residues such as R13 and K16 can still cover all four domains consistent with the observation that *µ*-GIIIA[K11A] can also block the channel [53]. The fact that two basic residues are sufficient to block the pore is also supported by several other *µ*-conotoxin blockers of Na_V_1 channels, which have only two basic residues available at the binding interface (e.g., BuIIIB and SIIIA).

Compared to potassium channels there are relatively fewer blockers of sodium channels, which is due to the larger pore size in the latter. A pore inserting Lys is sufficient to block a potassium channel whereas at least two basic residues are required to achieve the same in a sodium channel. The pore inserting Lys motif has been instrumental in functional studies of potassium channels using toxins peptides as probes. This has simplified interpretation of experimental results as well as construction of computational models of channel–toxin complexes. The situation in Na_V_1 channels is much more complicated due to many possible configurations for coupling of 2–3 basic toxin residues with the EEDD residues in the pore. Also there are many high-affinity toxins that do not block Na_V_1 channels. These features have certainly made interpretation of mutation experiments a more difficult task and sometimes resulted in conflicting proposals for the binding modes. Construction of accurate complex models using homology models of Na_V_1 channels is expected to ameliorate this situation. Moreover such complex models will be very useful in designing analogues with enhanced affinity and selectivity properties, which may be required for development of toxin blockers of Na_V_1 channels as therapeutic agents.

### Umbrella Sampling Simulations and Binding Free Energy

**Figure 5:**
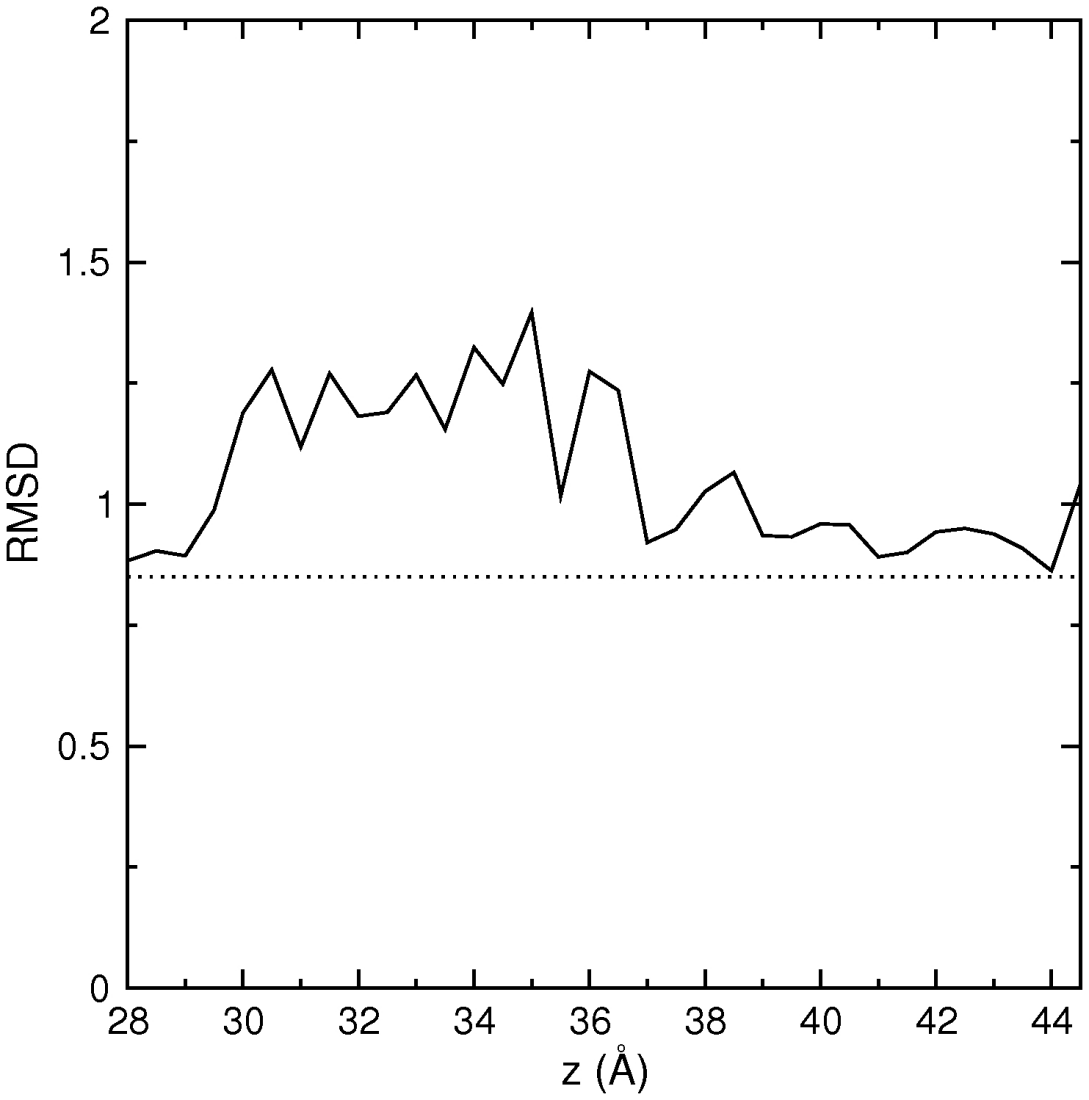
Average rmsd of the *µ*-GIIIA backbone atoms for the residues 11–22 calculated for each umbrella window. The bulk value obtained from MD simulations of *µ*-GIIIA in a box of water is indicated by the dashed line.

We have previously shown in over a dozen case studies involving potassium channel toxins that the binding free energy can be determined near chemical accuracy from PMF calculations [58]–[63]. Thus calculation of the standard binding free energy of *µ*-GIIIA will provide a complementary test for the accuracy of the proposed Na_V_1.4–*µ*-GIIIA model. The PMF for the dissociation of *µ*-GIIIA from Na_V_1.4 is constructed from umbrella sampling MD simulation as described in Methods. Distortion of a ligand during umbrella sampling simulations is a concern in PMF calculations, especially for flexible peptides, which may lead to erroneous results if the distortion becomes permanent after the ligand [65]. We have checked against this possibility by calculating the average rmsd of *µ*-GIIIA in each umbrella sampling window. As shown in Figure 5, the toxin undergoes some distortion while it is pulled out of the binding pocket but the elastic energy associated with this distortion is recovered once the toxin is in the bulk region as indicated by the return of the rmsd to the bulk value. Lack of distortion is also verified by aligning the *µ*-GIIIA structures from the last umbrella window with the NMR structure.

**Figure 6:**
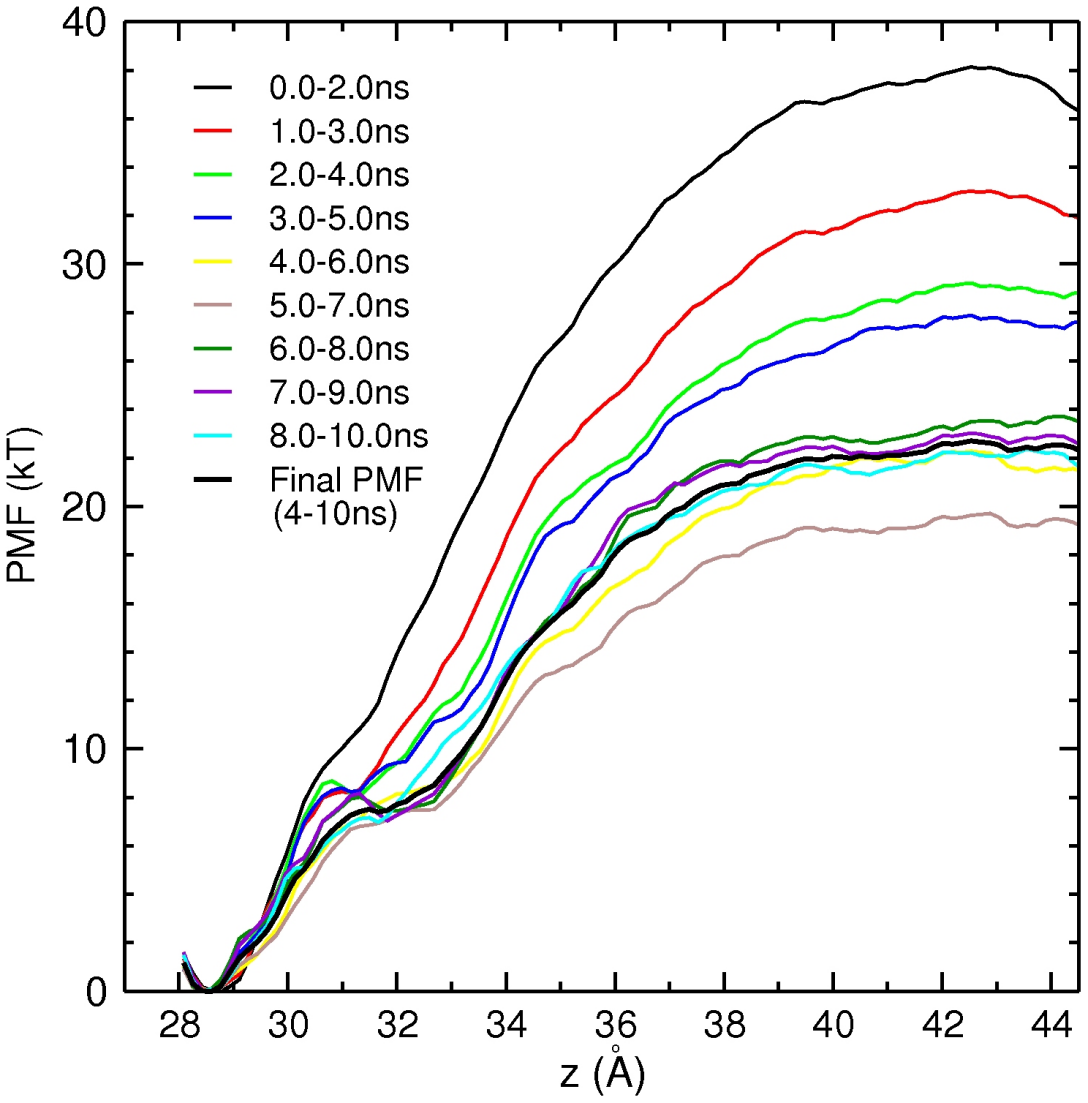
Convergence study of the Na_v_1.4–*µ*-GIIIA PMF from block data analysis. To minimize fluctuations, a relatively large sampling size of 2 ns is used, which is slided in 1 ns steps over the 10 ns of data. The block-data PMFs drop monotonically in the first 4 ns as the system equilibrates and then fluctuate around a base line from 4–10 ns, signalling equilibration. The final PMF obtained from 4–10 ns is indicated by a thick black line.

Two main questions in PMF calculations are how far the PMF should be extended and how long each window should be run. The first is addressed by appearance of a flat region in the PMF which indicates that the ligand has reached the bulk region. The second question is rarely addressed in PMF calculations but it is equally important because without sufficient data, one is likely to obtain a wrong answer. We address this issue by performing block data analysis of the PMF data. That is, we construct PMFs from 2 ns blocks of data and slide the blocks in 1 ns steps over the range of the available data. As shown in Figure 6, the PMFs initially drop monotonically and then fluctuate around a base line. During the first phase (1–4 ns), the PMFs drop because of the improved screening of the channel–toxin interactions as the system equilibrates. In the second phase (4–10 ns), fluctuations of the PMFs around a base line are of statistical nature and indicate that the system has been equilibrated. A common practice in PMF calculations is to exclude an arbitrary amount of data for equilibration (e.g., 1 ns), and consider the rest of the data in production of the PMF. As seen from the convergence study in Figure 6, this could result in mixing of the equilibration and production data, which would lead to an overestimation of the binding affinity.

The final PMF for the Na_V_1.4–*µ*-GIIIA complex is indicated by a thick black line in Figure 6. We will comment on distinct features of the PMF when we discuss the evolution of the distances between the contact pairs below. Using Eq. 1, we numerically integrate the PMF to find the binding constant and then determine the standard binding free energy using Eq. 2. The calculated value, 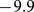 kcal/mol, is in good agreement with the experimental value of 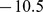 kcal/mol, which is determined from the most recent IC_50_ measurement (19 nM) where state-of-the-art equipment was employed [74]. The level of agreement obtained for the standard binding free energy is similar to those obtained for potassium channel toxins [58]–[64], and provides further support for the accuracy of the proposed model of the Na_V_1.4–*µ*-GIIIA complex.

**Figure 7:**
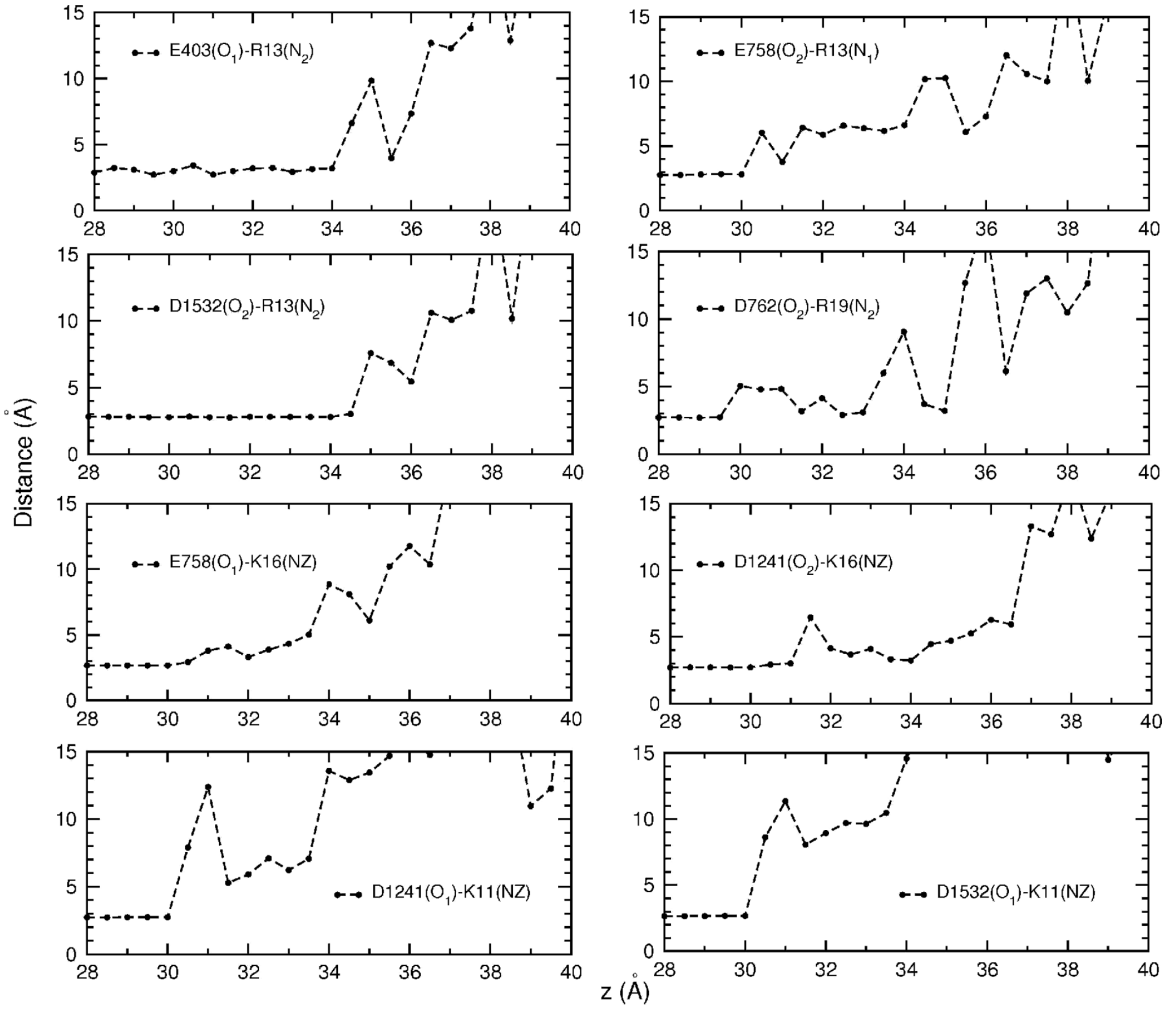
Evolution of the distances between the interacting pairs as *µ*-GIIIA dissociates from Na_v_1.4. Average N–O distances obtained from each umbrella window are plotted as a function of the window position. All the pairs in Table 2 are considered except K8–D1248 which dissociates immediately.

The binding mode of the Na_V_1.4–*µ*-GIIIA complex is dominated by charge interactions where the pairs are mostly at contact distances (Table 2). Analysis of how the contact distances change during dissociation provides complementary information on the relative strength of the various interactions as well as helping to explain specific features of the PMF. For this purpose, we have calculated the average pair distances in each umbrella window and plotted them as a function of the window position (Figure 7). The toxin residues are seen to break up in the following order and at the approximate positions: K11 at 30 Å, K16 at 34 Å, R19 at 35 Å, and finally R13 at 36 Å. Because the weakest interactions will break up first and the strongest ones last, the persistence length of a pair during dissociation provides a good measure for its relative strength. This intuitive picture for the strength of the interactions is in good agreement with the mutation data in Table 2. For example, according to the set of data from [53], the affinity loss for mutations of K11, K16, R19, and R13 to Ala are, respectively, 10, 81, 199, and 642.

Apart from the glitch around 31–32 Å, the final PMF in Fig 6 follows a steadily rising trajectory until all the contact pairs are broken off at 36 Å. The glitch appears to be associated with the fluctuations of the K11 and K16 pair distances (Figure 7). After 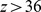 Å, the charged pairs interact via screened Coulomb interactions which corresponds to the shoulder region in the PMF. For 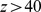 Å, the distances between the charged pairs are larger than 15 Å. At those distances, the Coulomb interactions are mostly screened off, which correlates well with the PMF leveling off after 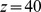 Å and becoming flat for 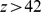 Å (Figure 6).

## Conclusions

In this work, we have constructed a homology model for the pore domain of the Na_V_1.4 channel by aligning the DEKA residues in the selectivity filter with the corresponding EEEE residues in Na_V_Ab, whose crystal structure was determined recently. In order to validate the Na_V_1.4 model, we have used the extensive functional data obtained from binding studies of *µ*-conotoxin GIIIA. An initial model for the Na_V_1.4–*µ*-GIIIA complex is created using HADDOCK, which is refined in MD simulations. The binding mode obtained is in broad agreement with the available mutagenesis data and shows that the toxin blocks the pore through multiple interactions of the R13, K16 and K11 residues with the outer ring of EEDD residues in the channel vestibule. The standard binding free energy of *µ*-GIIIA is determined from the PMF calculations and found to agree with the experimental value within chemical accuracy. Thus the proposed model of the Na_V_1.4–*µ*-GIIIA complex has been well validated. Because there is a high degree of homology among the Na_V_1 channels, the present Na_V_1.4 model can be used as a template in constructing homology models for the pore domain of other Na_V_1 channels.

Our focus in this first study was the validation of the pore domain using the data on binding of *µ*-GIIIA. For further studies of toxin binding to Na_V_1 channels one needs to include the selectivity filter and the S5-P1 linker in the model. For example, to investigate the ionic strength dependence of toxin binding [54], a validated model of the selectivity filter is required. This can be achieved by studying the permeation and selectivity properties of Na^+^ ions, which we hope to tackle in a forthcoming paper. Our attempts to model the S5-P1 linker in the turret of the Na_V_1.4 channel have not been successful due to lack of good templates. Because binding of *µ*-GIIIA does not appear to involve the S5-P1 linker residues, a satisfactory binding mode could still be obtained without modeling this region. However, the S5-P1 linker residues are involved in binding of some other *µ*-conotoxins, and to understand the differences in their affinity and selectivity properties [74], it will be important to construct models of Na_V_1 channels including the full turret region. The available mutagenesis data could provide valuable guidance in this endeavor. Finally, there are ongoing efforts to harness the therapeutic potential of *µ*-conotoxins by designing analogues that selectively bind to a target Na_V_1 channel [3]–[5]. Construction of accurate models of Na_V_1–*µ*-conotoxin complexes will be very useful in such efforts.

After the completion of this work, a paper on binding of *µ*-conotoxin PIIIA to the Na_V_1.4 channel has appeared [75]. In this paper, two very different binding modes were predicted with almost equal binding free energies. This is very different from the unique binding mode found for *µ*-GIIIA in our study and needs to be investigated further. A snapshot comparing the Na_V_1.4 models used in the two studies is given in Figure S1 in File S1.

## Supporting Information

File S1 Figure S1, Snapshot comparing the pore domain of our Nav1.4 model with that of Chen et al. [75].
(PDF)

File S2 PDB file giving the coordinates of the Nav1.4-GIIIA complex model used in drawing Figure 3.
(PDB)
