# Supplementary material for: Molecular Dynamics Study of Binding of µ-Conotoxin GIIIA to the Voltage-Gated Sodium Channel Nav1.4
Source: PLoS One. 2014 Aug 18;9(8):e105300. doi: 10.1371/journal.pone.0105300 (PMC4136838; doi:10.1371/journal.pone.0105300)
Supplement: File S1 — Figure S1, Snapshot comparing the pore domain of our Nav1.4 model with that of Chen et al. [75]. (PDF) [file pone.0105300.s001.pdf]

Supporting Information for
Molecular Dynamics Study of Binding of μ-Conotoxin GIIIA to the Voltage-Gated Sodium Channel $Na_V1.4$

Somayeh Mahdavi and Serdar Kuyucak

School of Physics, University of Sydney, New South Wales 2006, Australia

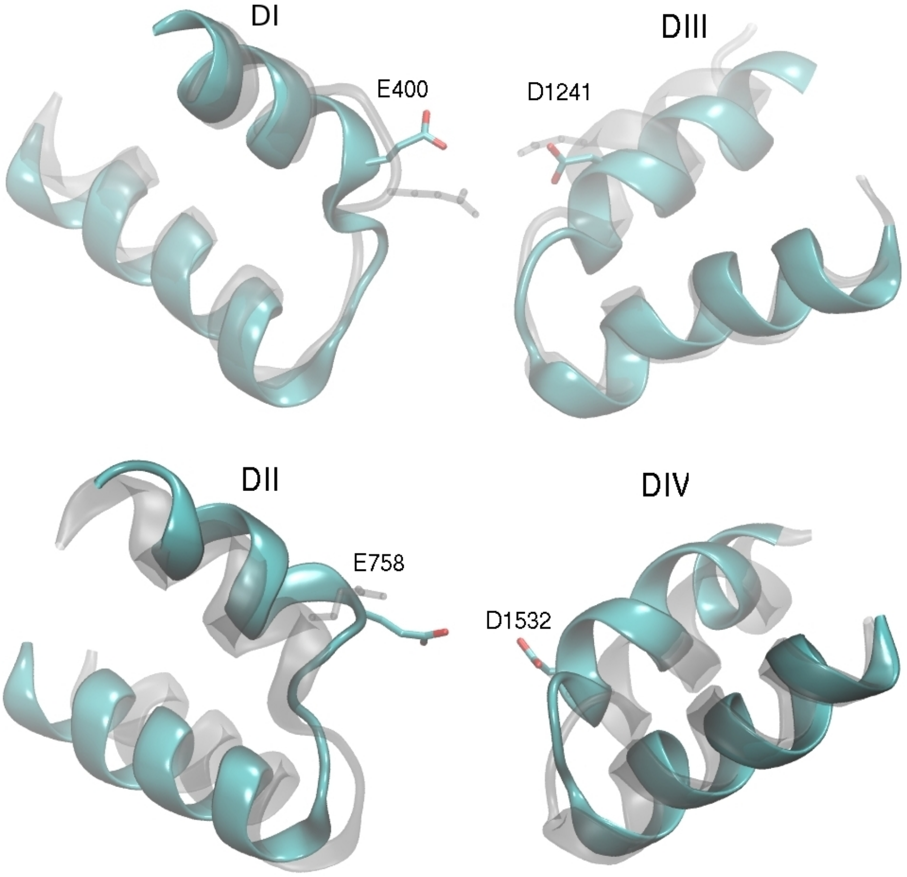

Figure 1: Snapshot comparing the pore domain of our Nav1.4 model with that of Chen et al. [75].
